# Impact of functional status on 30-day resource utilization and organ system complications following index bariatric surgery: a cohort study

**DOI:** 10.1097/JS9.0000000000000785

**Published:** 2023-09-22

**Authors:** Z. Logan Holley, Ziyad O. Knio, Long-Quan Pham, Unique Shakoor, Zhiyi Zuo

**Affiliations:** a School of Medicine, University of Virginia; b Department of Anesthesiology, University of Virginia Health, Charlottesville, VA, USA

**Keywords:** bariatric surgery, dependent functional status, frailty, postoperative morbidity, risk stratification, surgical complications

## Abstract

**Background:**

Bariatric surgical procedures carry an appreciable risk profile despite their elective nature. Identified risk factors for procedural complications are often limited to medical comorbidities. This study assesses the impact of functional status on resource utilization and organ system complications following bariatric surgery.

**Materials and methods:**

This retrospective cohort study analyzed patients undergoing elective, index bariatric surgery from American College of Surgeons National Surgical Quality Improvement Program participating hospitals from 2015 to 2019 (*n*=65 627). The primary independent variable was functional status. The primary outcome was unplanned resource utilization. Secondary outcomes included composite organ system complications and mortality. The impact of functional status was first investigated with univariate analyses. Survival and multivariate analyses were then performed on select complications with clinically and statistically significant incidence in the dependent cohort.

**Results:**

On univariate analysis, dependent functional status was associated with unplanned resource utilization [12.1% (27/223) vs. 4.1% (2661/65 404)]; relative risk, 2.98 (95% CI, 2.09–4.25); *P* < 0.001] and haematologic/infectious complications [6.7% (15/223) vs. 2.4% (1540/65 404); relative risk, 2.86 (95% CI, 1.75–4.67); *P* < 0.001]. Survival analysis demonstrated a significantly shorter time to both events in patients with dependent functional status (*P* < 0.001). On multivariate analysis, dependent functional status was an independent predictor of unplanned resource utilization[adjusted odds ratio 2.17 (95% CI, 1.27–3.50); *P* = 0.003; model c-statistic, 0.572]) and haematologic/infectious complications [adjusted odds ratio, 2.20 ([95% CI, 1.14–3.86); *P* = 0.011; model c-statistic, 0.579].

**Conclusion:**

Patients with dependent functional status are at an elevated risk of unplanned resource utilization and haematologic/infectious complications following index bariatric surgery. The increased risk cannot be explained by medical comorbidities alone.

## Introduction

Highlights

- The effect of functional status on bariatric surgical complications remains unclear.
- Dependent functional status is associated with unplanned resource utilization and haematologic/infectious complications.
- Multivariate and survival analyses support the above findings.
- Unweighted risk scores can predict unplanned resource utilization and haematologic/infectious complications after bariatric surgery.

Patient frailty has gained considerable attention as a potential predictor of morbidity and mortality following surgical procedures and during inpatient hospitalizations^1–3^. Recently this subjective patient state has been represented by an eleven-point “frailty index” to promote its generalizability^4,5^. Unfortunately frailty indices are subject to influence by missing variables, and they are often discretized to aid in interpretability^2,3,6^. Functional dependence is just one component of patient frailty, yet it has been shown to be a predictor of 30-day mortality within each American Society of Anesthesiologists class^7^. Functional status may therefore serve as a less cumbersome estimate of overall frailty, and its association with surgical outcomes may help to inform patients of their overall perioperative risk.

The bariatric surgical patient population is unique in that the scheduled surgery is elective yet associated with a non-insignificant risk profile^8,9^. Risk factors for various outcomes, such as readmission, morbidity, and mortality, have been studied extensively; however, the identified risk factors are often limited to medical comorbidities, major postoperative complications, or socioeconomic determinants of health^10–14^. The modest incidence of postoperative complications therefore makes this population ideal for investigating the relationship between functional status and adverse perioperative outcomes. Recent studies have suggested that in the bariatric surgical population, functional dependence is associated with increased rates of major morbidity, mortality^14–16^, and readmission^17^.

This study was designed to investigate whether functional status was associated with adverse events following index bariatric surgery. The authors hypothesized that dependent functional status would be associated with increased 30-day resource utilization events, among other 30-day organ systemic complications.

## Material and methods

### Study design and data sources

This research was retrospectively registered with clinicaltrials.gov. This study was exempt from Institutional Review Board approval given that it was a retrospective cohort analysis of a national de-identified database. Cases were obtained from the American College of Surgeons National Surgeons Quality Improvement Program (ACS-NSQIP) participant use data file. This is a prospective, validated multicenter surgical outcomes database. The user guides specify that “the ACS-NSQIP collects data on over 150 variables, including preoperative risk factors, intraoperative variables, and 30-day postoperative mortality and morbidity outcomes for patients undergoing major surgical procedures in both the inpatient and outpatient setting.” Functional status is one field within the data file. Additional details regarding specific variable definitions, internal validation and sampling selection can be found in the user guide^18^.

The ACS-NSQIP database from 2015 to 2019 was queried for the following bariatric surgical procedures: gastric bypass, vertical or adjustable band, sleeve gastrectomy, and biliopancreatic diversion [Current Procedural Terminology (CPT) codes 43644, 43645, 43770, 43775, 43842, 43843, 43845, 43846, 43847]). When applicable, both laparoscopic and open approaches were included. The work has been reported in line with the STROCSS criteria^19^, Supplemental Digital Content 1, http://links.lww.com/JS9/B86.

### Patient inclusion and exclusion criteria

After the initial query, the following criteria were applied in order to produce a more homogenous investigation sample. Only elective, non-emergent, index cases were included. Cases with documented concurrent or other procedures were excluded. Cases were required to have a documented “general surgery” surgical specialty and “general anesthesia” anaesthetic technique. Patients admitted to the hospital for greater than one day preceding surgery were excluded. Patients with preoperative documentation of acute kidney injury, end-stage renal disease, metastatic disease, wound infection, and sepsis were excluded. Those experiencing these diagnoses postoperatively were not excluded. These criteria were agreed upon by the study authors *a priori*.

### Measurements and data handling

The primary independent variable was functional status. This was dichotomized as “independent” for those documented as such, and “dependent” for those documented as “partially dependent” or “totally dependent” in the ACS-NSQIP database. While current user files (2015 and later) did not clarify this variable’s standardization, earlier user files (2014 and prior) defined independence as an individual’s ability to complete activities of daily living with or without prosthetics, equipment, or devices, but explicitly without the assistance of another individual^18^. Patients with unknown functional status were excluded from analysis, in accordance with the study objectives.

Independent variables also included age, sex, BMI, hypertension, insulin-dependent diabetes, currently smoking, chronic obstructive pulmonary disease (COPD), congestive heart failure (CHF), chronic steroid use, and bleeding disorders.

The following preoperative serum laboratory results were also considered: sodium concentration, blood urea nitrogen concentration, creatinine concentration, albumin concentration, bilirubin concentration, aspartate aminotransferase concentration, alkaline phosphatase concentration, white blood cell count, haematocrit (%), and platelet count.

The primary outcome was 30-day unplanned resource utilization, which included unplanned readmission, return to the operating room, and prolonged (≥ 30 days) length of stay.

Secondary outcomes included 30-day composite organ system complications and 30-day mortality. Cardiac complications included cardiac arrest and myocardial infarction. The only neurologic complication that was captured was stroke/cerebrovascular accident. Respiratory complications included reintubation, prolonged ventilatory wean, and pneumonia. Haematologic/infectious complications included superficial surgical site infection (SSI), deep incisional SSI, organ space SSI, wound dehiscence, bleeding, sepsis, septic shock, deep venous thrombosis, and pulmonary embolism. Renal complications included progressive renal insufficiency and acute kidney injury.

### Statistical analysis

Statistical analysis was performed with R version 4.2.0 (R Core Team)^20^. Continuous variables were summarized by mean (standard deviation), while categorical variables were summarized by frequency (%). All hypothesis tests were two-sided, with significance defined by α less than or equal to 0.05.

Associations between functional status and all of the above outcomes were investigated with univariate analyses. Fisher’s exact test was applied due to the anticipated low event rate for 30-day complications. Additionally, relative risks (RR) and fragility indices were calculated following Fisher’s exact methodology^21^.

Survival analyses were then performed on select composite outcomes with clinically (*n* ≥ 10) and statistically (α ≤ 0.05) significant incidence in the dependent functional status cohort after verifying a robust fragility index. Kaplan–Meier curves modelling days of event-free survival against functional status were constructed. The risk attributable to dependent functional status was quantified with the log-rank *P* value^22,23^.

Multivariate analyses were also performed on the select composite outcomes. Multiple logistic regression modelling was applied, with adjustments made for age, sex, BMI, hypertension, diabetes, smoking, COPD, CHF, chronic steroid use, and bleeding disorders when significance was demonstrated on univariate testing at α less than or equal to 0.05. Adjustments were also made for preoperative haematologic and metabolic serum laboratory results; however, the serum markers were subjected to a Bonferroni-adjusted significance of α less than or equal to 0.005 in order to minimize the error introduced by multiple comparisons and to avoid potential model overfitting. Only complete cases were considered, and missing data were not imputed. Subsequent variable selection was accomplished by backwards stepwise model adjustment by Akaike information criterion. Adjusted odds ratios (AOR) and accompanying confidence intervals are reported for the independent predictors of the select composite outcomes^24^. Weighted and unweighted risk scores were constructed and their performance relative to the multivariate model was validated with receiver operating characteristic analysis^25^.

## Results

### Demographics and medical comorbidities

Of 65 627 bariatric surgical patients meeting inclusion criteria, there were 223 (0.3%) with dependent functional status. The mean age of the study population was 43.3 (11.9) years and mean BMI was 45.5 (8.0) kg/m^2^. A majority of the patients included in the study were female (75.9%). Of the 65 627 patients, 29 410 (44.8%) had a diagnosis of hypertension, 5240 (8.0%) had insulin-dependent diabetes, 5761 (8.8%) had active smoking history, 965 (1.5%) had COPD, 241 (0.4%) had CHF, 1176 (1.8%) were on chronic steroids, and 559 (0.9%) had a bleeding disorder. Compared to functionally independent individuals, those with some degree of dependence were older, of greater BMI, and more likely to be male or have hypertension, insulin-dependent diabetes, COPD, CHF, chronic steroid use, or bleeding disorders (Table 1).

**Table 1:** Demographic comparisons by functional status classification.

	Mean (SD) or *n*/total (%)			
Characteristic	Independent *n* = 65 404	Dependen*t n* = 223	Mean difference or odds ratio [95% CI]	*P*
Age (years)	43.3 (11.9)	51.2 (11.9)	7.93 [6.36–9.51]	<0.001
BMI (kg/m^2^)	45.5 (7.9)	52.4 (12.5)	6.95 [5.30–8.61]	<0.001
Sex (female)	52 043/65 402 (79.6)	159/223 (71.3)	0.64 [0.48–0.85]	0.003
Hypertension	29 263/65 404 (44.7)	147/223 (65.9)	2.38 [1.81–3.14]	<0.001
Insulin-dependent diabetes	5193/65 404 (7.9)	47/223 (21.1)	3.08 [2.23–4.24]	<0.001
Current smoker	5740/65 404 (8.8)	21/223 (9.4)	1.08 [0.69–1.69]	0.722
COPD history	942/65 404 (1.4)	23/223 (10.3)	7.71 [5.03–11.81]	<0.001
CHF history	233/65 404 (0.4)	8/223 (3.6)	10.10 [5.04–20.21]	<0.001
Chronic steroid use	1166/65 404 (1.8)	10/223 (4.5)	2.57 [1.37–4.84]	0.007
Bleeding disorder	546/65 404 (0.8)	13/223 (5.8)	7.21 [4.14–12.54]	<0.001

CHF, congestive heart failure; COPD, chronic obstructive lung disease.

### Univariate analyses by functional status

Dependent functional status was associated with unplanned resource utilization [RR, 2.98 (95% CI, 2.09–4.25); *P* < 0.001], cardiac complications [RR, 11.73 (95% CI, 3.73–36.92)]; *P* = 0.002], respiratory complications [RR, 7.49 (95% CI, 3.37–16.66); *P* < 0.001], haematologic/infectious complications [RR, 2.86 (95% CI, 1.75–4.67); *P* < 0.001], and renal complications [RR, 6.67 (95% CI, 1.65–26.9); *P* = 0.038]. Dependent functional status was not associated with mortality or neurologic complications, of note the event rate for both outcomes in the dependent functional status cohort was 0/223 (Table 2).

**Table 2:** Univariate analyses on 30-day outcomes by functional status classification.

	*N*/total (%)				
Outcome	Independent *n* = 65 404	Dependent *n* = 223	Relative risk [95% CI]	*P*	Fragility index
Unplanned resource utilization (composite)	2661/65 404 (4.1)	27/223 (12.1)	2.98 [2.09–4.25]	<0.001	12
Unplanned readmission	2246/65 404 (3.4)	24/223 (10.8)	3.13 [2.14–4.58]	<0.001	11
Unplanned reoperation	865/65 404 (1.3)	9/223 (4.0)	3.05 [1.60–5.81]	0.003	3
>30-day length of stay	41/65 404 (0.1)	0/223 (0.0)	0.00 [0.00–NaN]	>0.999	2
Cardiac complications (composite)	75/65 404 (0.1)	3/223 (1.3)	11.73 [3.73–36.92]	0.002	2
Cardiac arrest	42/65 404 (0.1)	0/223 (0.0)	0.00 [0.00–NaN]	>0.999	2
Myocardial infarction	37/65 404 (0.1)	3/223 (1.3)	23.78 [7.39–76.56]	<0.001	2
Neurologic complications (stroke)	10/65 404 (0.0)	0/223 (0.0)	0.00 [0.00–NaN]	>.999	1
Respiratory complications (composite)	235/65 404 (0.4)	6/223 (2.7)	7.49 [3.37–16.66]	<0.001	4
Unplanned reintubation	79/65 404 (0.1)	4/223 (1.8)	14.85 [5.49–40.20]	<0.001	3
>48 h ventilator dependence	66/65 404 (0.1)	3/223 (1.3)	13.33 [4.22–42.08]	0.002	2
Pneumonia	146/65 404 (0.2)	2/223 (0.9)	4.02 [1.00–16.12]	0.091	1
Haematologic/infectious complications (composite)	1540/65 404 (2.4)	15/223 (6.7)	2.86 [1.75–4.67]	<0.001	6
Superficial SSI	449/65 404 (0.7)	4/223 (1.8)	2.61 [0.99–6.93]	0.070	1
Deep incisional SSI	45/65 404 (0.1)	2/223 (0.9)	13.04 [3.18–53.40]	0.011	1
Organ space SSI	263/65 404 (0.4)	2/223 (0.9)	2.23 [0.56–8.91]	0.228	2
Wound dehiscence	53/65 404 (0.1)	1/223 (0.4)	5.53 [0.77–39.84]	0.168	1
Bleeding/transfusion	487/65 404 (0.7)	5/223 (2.2)	3.01 [1.26–7.20]	0.027	1
Sepsis	155/65 404 (0.2)	3/223 (1.3)	5.68 [1.82–17.66]	0.017	1
Septic shock	65/65 404 (0.1)	1/223 (0.4)	4.51 [0.63–32.37]	0.201	1
Deep venous thrombosis	192/65 404 (0.3)	1/223 (0.4)	1.53 [0.22–10.85]	0.482	2
Pulmonary embolism	97/65 404 (0.1)	1/223 (0.4)	3.02 [0.42–21.59]	0.284	1
Renal complications (composite)	88/65 404 (0.1)	2/223 (0.9)	6.67 [1.65–26.91]	0.038	1
Acute kidney injury	37/65 404 (0.1)	2/223 (0.9)	15.85 [3.84–65.38]	0.008	1
Progressive renal insufficiency	51/65 404 (0.1)	0/223 (0.0)	0.00 [0.00–NaN]	>0.999	2
Mortality	48/65 404 (0.1)	0/223 (0.0)	0.00 [0.00–NaN]	>0.999	2

NaN, not a number; SSI, surgical site infection.

### Survival analyses by functional status

**Figure 1:**
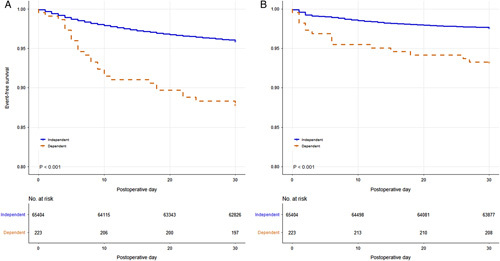
Survival analysis by functional status for (A) unplanned resource utilization events and (B) haematologic/infectious complications.

There was a clinically (*n* > 10) and statistically (*P* < 0.050) significant incidence of unplanned resource utilization and haematologic/infectious complications in the dependent functional status cohort. Fragility indices for unplanned resource utilization and haematologic/infectious complications were 12 and 6, respectively (Table 2). Survival analysis demonstrated a significantly shorter time to unplanned resource utilization and haematologic/infectious complications in patients with dependent functional status (Fig. 1).

### Multivariate analyses by outcome

On multivariate analysis, dependent functional status was independently predictive of unplanned resource utilization events [AOR, 2.17 (95% CI, 1.27–3.50); *P* = 0.003] and haematologic/infectious complications [AOR, 2.20 (95% CI, 1.14–3.86); *P* = 0.011], after adjusting for relevant covariates. It predicted unplanned resource utilization events with greater odds than BMI, hypertension, insulin-dependent diabetes, current smoking status, COPD, chronic steroid use, or bleeding disorders, but lesser odds than CHF [AOR, 2.42 (95% CI, 1.50–3.72); *P* < 0.001] (model c-statistic: 0.572). It predicted haematologic/infectious complications with greater odds than BMI, insulin-dependent diabetes, COPD, chronic steroid use, but lesser odds than bleeding disorders [AOR, 2.26 (95% CI, 1.47–3.32); *P* < 0.001] (model c-statistic: 0.579) (Table 3).

**Table 3:** Multivariate analyses with unweighted risk scores for select 30-day outcomes predictions.

	Unplanned resource utilization events			Haematologic/infectious complications		
Characteristic	Adjusted odds ratio [95% CI]	Adjusted *P* value	Unweighted risk score	Adjusted odds ratio [95% CI]	Adjusted *P* value	Unweighted risk score
Age^a^ >50 years				1.01 [1.01–1.02]	<0.001	+1
BMI^a^ >50 kg/m^2^	1.01 [1.00–1.01]	0.029	+1	1.01 [1.00–1.02]	0.047	+1
Hypertension	1.10 [1.00–1.22]	0.056	+1			
Insulin-dependent diabetes	1.42 [1.22–1.65]	<0.001	+1	1.20 [0.98–1.46]	0.076	+1
Current smoker	1.13 [0.97–1.32]	0.115	+1			
COPD history	1.78 [1.33–2.32]	<0.001	+1	2.10 [1.49–2.87]	<0.001	+1
CHF history	2.42 [1.50–3.72]	<0.001	+1			
Chronic steroid use	1.33 [0.98–1.76]	0.055	+1	1.48 [1.02–2.07]	0.029	+1
Bleeding disorder	1.94 [1.36–2.70]	<0.001	+1	2.26 [1.47–3.32]	<0.001	+1
Dependent functional status	2.17 [1.27–3.50]	0.003	+1	2.20 [1.14–3.86]	0.011	+1
Sodium^a^ <135 mmol/L	0.97 [0.95–0.99]	<0.001	+1			
Creatinine^a^ >1.5 mg/dL	1.09 [0.97–1.21]	0.120	+1			
Albumin^a^ <3.5 g/dL	0.82 [0.72–0.93]	0.003	+1	0.76 [0.64–0.89]	0.001	+1
c-statistic	0.572		0.560	0.579		0.563
*n* ^b^	41 466			42 831		

a The adjusted odds ratio and adjusted *P* value reported for these continuous variables in fact summarizes the non-discrete characteristic (i.e. age in years), while the unweighted risk score value is calculated using the discretized characteristic (i.e. +1 for age >50).

b Cases with missing data were excluded from multivariate analysis.

The addition of each risk factor increased the incidence of resource utilization events, as follows: 0, 3.5%; 1, 3.9%; 2, 5.2%; 3, 6.8%; 4, 8.3%; 5, 15.1%; 6+, 16.0%.

The addition of each risk factor increased the incidence of haematologic/infectious complications, as follows: 0, 2.0%; 1, 2.5%; 2, 3.4%; 3, 4.7%; 4, 8.9%; 5+, 10.7%.

CHF, congestive heart failure; COPD, chronic obstructive lung disease.

### Risk score analysis

**Figure 2:**
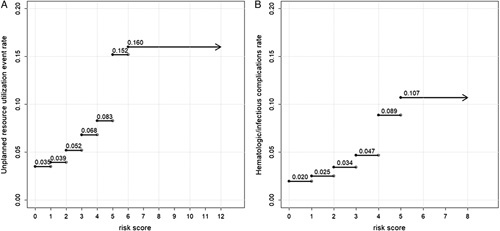
Stepwise plot function of event incidence against risk score for (A) unplanned resource utilization and (B) haematologic/infectious complications.

**Figure 3:**
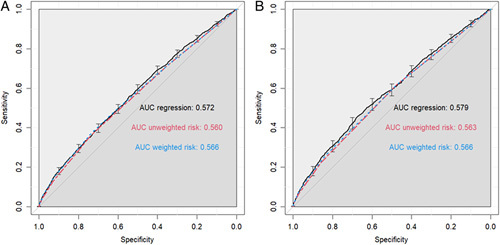
Receiver operating characteristic curves for (A) resource utilization events risk scoring and (B) haematologic/infectious complications risk scoring. AUC, area under the curve.

Quantitative predictors in the multiple logistic regression models were discretized following receiver operating characteristic investigation, and risk scores were constructed. Weighted risk scores were initially considered; however, their performance was not superior to those of simplified, unweighted risk scores. Therefore the unweighted risk scores were favored due to ease-of-use. The unweighted risk score for unplanned resource utilization events was equal to the sum of one point for each of the following: dependent functional status, BMI greater than 50 kg/m^2^, hypertension, insulin-dependent diabetes, being a current smoker, COPD, CHF, chronic steroid use, bleeding disorders, sodium less than 135 mmol/l, creatinine greater than 1.5 mg/dl, and albumin less than< 3.5 g/dl (c-statistic: 0.560). The unweighted risk score for haematologic/infectious complications was equal to the sum of one point for each of the following: dependent functional status, age older than 50 years, BMI greater than 50 kg/m^2^, insulin-dependent diabetes, COPD, chronic steroid use, bleeding disorders, and albumin less than 3.5 g/dl (c-statistic: 0.563) (Table 2). Higher unweighted risk scores reliably corresponded to an increased adverse event rate (Fig. 2). The respective receiver operating characteristic curves for the unweighted risk scores were contained within the 95% confidence interval of the corresponding multiple logistic regression models prior to discretization; the performance of the weighted, unweighted, and logistic regression risk scores is summarized in Fig. 3 and eTables 1-2 (Supplemental Digital Content 1, Supplemental Digital Content 2, http://links.lww.com/JS9/B87).

## Discussion

This study determined that functional status was not only associated with significant medical and surgical complications following bariatric surgery, but it carried a significant added-risk for unplanned resource utilization events and haematologic/infectious complications. It was the single most influential predictor of haematologic/infectious complications, and the second most influential predictor of unplanned resource utilization. Interestingly, serum albumin concentration demonstrated a strong association with both adverse events. Functional status was the primary independent variable in this study, as it was thought to be a binary and unambiguous proxy for patient frailty.

Functional status has been investigated as a potential predictor of complications following a variety of ACS-NSQIP indexed surgeries including colectomy, thyroidectomy, low anterior resection or abdominoperineal resection of colorectal cancer, surgery for spinal tumours, thoracic surgery, craniotomy for tumour, and total hip arthroplasty^26–32^. The existing studies have concluded that preoperatively functionally dependent patients are at higher risk for major morbidity and mortality.

Lak *et al.* ^14^ investigated 148 710 minimally invasive bariatric surgical patients and demonstrated that short-term morbidity and mortality risk increased significantly in functionally dependent patients. Of note, the prevalence of dependent functional status in their study was greater than that of the present study (1.0% vs. 0.3%). Khorgami *et al.* ^17^ investigated laparoscopic Roux-en-Y gastric bypass and laparoscopic sleeve gastrectomy cases in the ACS-NSQIP database and demonstrated that dependent functional status was one independent predictor of readmission [AOR, 1.94 (95% CI, 1.06–3.55), *P* = 0.032]. This is concordant with the 2.17 adjusted odds of unplanned resource utilization determined in the present study.

The present study is strengthened by discretization of outcomes according to organ system, rather than constructing a singular composite endpoint to capture all-cause morbidity and mortality^33–35^. Discretization methodology in previous studies has been variable. Gupta *et al.* ^36^ assessed patients undergoing bariatric surgery for morbid obesity with a primary outcome of major morbidity due to 17 postoperative complications that spanned multiple organ systems. Hornock *et al.* ^37^ further discretized morbidities in a similar investigation of revision bariatric surgeries into “major systemic”, “minor systemic”, “major local”, and “minor local” complications. Lak *et al.* ^14^ discretizes morbidities by organ system. This study is further strengthened by reporting of fragility indices for all endpoints, supporting the need for composite endpoints and validating the subsequent investigations into select outcomes^21^. Additionally, survival analysis confirmed that the time to adverse events was shorter in those with dependent functional status.

The methodology applied for risk score analyses is similar to what has been done previously. The weighted risk scores were constructed following the methodology outlined by Canet *et al.* ^38^ in the ARISCAT score for postoperative pulmonary complications. The unweighted risk score was designed to be easily applicable, much like the revised cardiac risk index initially described by Goldman and colleagues and the ANESCARDIOCAT model by Sabaté and colleagues^39,40^. Of note, the c-statistics of the present risk scores (0.560 and 0.563 for unplanned resource utilization events and haematologic/infectious complications, respectively) are less robust than those of the ARISCAT (0.89 and 0.84 for development and validation subsamples, respectively), the revised cardiac risk index (0.76), and the ANESCARDIOCAT (0.759)^38–40^. As such, the accuracy with which unplanned resource utilization and haematologic/infectious complications can be predicted using these risk scores is limited. However, the limitations of relying on c-statistics alone to assess model fit are well described^41^. Independent predictors of resource utilization events and haematologic/infectious complications are better determined by stepwise assessment of the Akaike information criterion, as was performed during model selection in the present study. Additionally, the present study demonstrated a stepwise increase in adverse event risk with the incremental addition of identified risk factors.

There are several important limitations that are inherent to the study design. This was an observational study, as such the defined outcomes and covariates were limited to those captured by the ACS-NSQIP database. The rare prevalence of functional dependence (0.3%) precluded the authors from performing meaningful subgroup analyses for each surgical approach. This limitation likely impacted a similar study population (with 0.4% functional dependence prevalence), where functional status was a quantified predictor of readmission in the aggregate cohort but not in laparoscopic gastric bypass vs. gastric sleeve subgroup analyses^17^. Adverse events were also observed in low frequencies; in fact, the most common adverse event was unplanned resource utilization (4.1% aggregate rate). Propensity score matching was not performed, as the adverse event rates even under a 1:2 schema (223 functionally dependent cases vs. 446 independent controls) would not be representative of those observed in a 65 627 unmatched sample. While an increased risk of adverse events was observed in the dependent functional status cohort both on the univariate and multivariate level, a causal relationship could not be determined. The potential benefit of exercise preconditioning or other preoperative interventions is yet to be defined^42,43^. The authors decided *a priori* to consider only complete cases in the multivariate analysis, in other words refraining from imputing missing data. While the multivariate samples contained an adequate sample size and event rate for logistic regression, sampling bias may have been introduced by excluding the presumably healthier patients that did not undergo preoperative laboratory testing^44–46^. The generalizability of the multivariate and risk scoring results may therefore be limited to patients in whom preoperative laboratory testing is either clinically indicated or institutional standard of practice. The univariate results are more generalizable to all index bariatric surgical patients.

## Conclusions

In summary, the present study demonstrates an increased risk of unplanned resource utilization and haematologic/infectious complications in bariatric surgical patients with dependent functional status. While routine preoperative serum laboratory testing may not be indicated in low-risk bariatric surgical patients, it should be obtained in those with functional dependence and other identified risk factors, such as COPD, CHF, and bleeding disorders. These markers would help to better risk stratify and optimize bariatric surgical patients. Those at high risk of haematologic/infectious complications may benefit from more aggressive perioperative blood management strategies.

## Ethical approval

Not needed; de-identified database study.

## Source of funding

This study is supported by Robert M. Epstein Professorship from the University of Virginia, Charlottesville, VA. The funder has no role in experimental design, data analysis, manuscript writing or decision on publication of this study.

## Author contribution

Z.L.H.: investigation, writing—original draft. Z.O.K.: methodology, software, formal analysis, writing—original draft. L.Q.P.: investigation, writing—original draft. U.S.: investigation, writing—original draft. Z.Z.: conceptualization, resources, writing—review and editing, supervision, project administration.

## Conflicts of interest disclosure

The authors declare that they have no known competing financial interests or personal relationships that could have appeared to influence the work reported in this paper.

## Guarantor

The ACS NSQIP database was accessed by Z.O.K. Z.O.K. and Z.Z. have full access to all the data in the study and take responsibility for the integrity of the data and the accuracy of the data analysis.

## Data availability statement

American College of Surgeons National Surgical Quality Improvement Program and the hospitals participating in the ACS NSQIP are the source of the data used herein. The Participant Use Data File (PUF) is a Health Insurance Portability and Accountability Act (HIPAA)-compliant data file containing cases submitted to the American College of Surgeons National Surgical Quality Improvement Program. To request a copy of the PUF, individuals (data recipients) must agree to comply with the terms and conditions set forth in the Data Use Agreement, provide contact information, and complete a short online questionnaire. Once the information provided by the data recipient is received and processed by ACS NSQIP staff, a website address will be submitted electronically to the data recipient. The data recipient will then have 10 days (240 hours) to visit the website and download the data file.

## Provenance and Peer Review

Not commissioned, externally peer-reviewed.
